# LASPAI-Assisted Mechanistic Investigation of Carbon Poisoning in Molecular Copper-Catalyzed CO_2_ Reduction

**DOI:** 10.3390/ma19214500

**Published:** 2026-10-26

**Authors:** Shanshan Lin, Guangjing Feng, Xiaoyan Sun

**Affiliations:** 1 State Key Laboratory of Photoelectric Conversion and Utilization of Solar Energy, Qingdao New Energy Shandong Laboratory, Qingdao Institute of Bioenergy and Bioprocess Technology, Chinese Academy of Sciences, Qingdao 266101, China; sunxy@qibebt.ac.cn; 2 University of Chinese Academy of Sciences, Beijing 100049, China; fengguangjing23@mails.ucas.ac.cn

**Keywords:** LASPAI, AI-based atomistic modeling, CO_2_ electroreduction, pulsed electrolysis, EC-ESI-MS

## Abstract

Strong retention of coordinated CO can divert molecular CO_2_ reduction from product release toward persistent carbon-containing species and catalyst deactivation. We examine this competition in a Cu(II)-derived Cudpk system using electrochemistry–electrospray ionization mass spectrometry (EC-ESI-MS), collision-induced dissociation (CID) and AI-assisted pathway simulation. Time-resolved EC-ESI-MS during the potential-pulse sequence detected Cudpk ions assigned to -–CO, –COH, –CHOH and –CH-associated compositions. In separate post-electrolysis solution samples, an *m*/*z* = 260 CH-associated ion was detected after constant-potential operation but was not obvious after approximately 16 h of pulsed operation. Double-ended surface walking (DESW) searches on LASPAI use a generalized global neural network (GGNN) potential to evaluate energies and forces. The resulting profile places a high energetic cost on CO release and identifies a competing route from retained CO toward a Cu-bound carbon state. Pulsed operation resolves this contradiction: periodic potential pulses suppress the accumulation of residual carbon-containing species, thereby maintaining the turnover of the CO formation reaction. The AI-derived energy profile and pulse-dependent mass spectrometry data support a proposed deactivation pathway involving further conversion of strongly retained CO into persistent carbon-associated species. The reduced persistence of CH-associated ions after pulsed operation, together with sustained CO production, is consistent with mitigation of this pathway.

## 1. Introduction

Molecular catalysts provide ligand environments in which CO_2_ activation, proton transfer and product release can be examined separately [1]. For catalysts producing CO, the lifetime and reactivity of the metal–CO state are important determinants of sustained turnover. In an iron tetracarbene heme analog, replacing an axial acetonitrile ligand with CO increases catalyst stability and turnover number [2]. In homogeneous nickel cyclam catalysis, by contrast, CO release from a deactivated carbonyl complex limits catalyst recovery, and a CO scavenger increases catalytic turnover [3]. The consequence of CO coordination therefore depends on the subsequent chemistry of the bound product: a metal–CO state can stabilize a catalyst, delay its return to the catalytic cycle, or provide a starting point for further conversion. Copper coordination chemistry provides a setting in which these possibilities can be investigated. More recently, systematic donor and macrocycle modifications of copper complexes have linked improved CO_2_-to-CO catalysis to stabilization of reactive intermediates and ligand redox reversibility [4]. The structures present during electrolysis can nevertheless differ from the prepared complexes. Copper phthalocyanine and benzimidazole-based systems undergo restructuring under CO_2_ reduction conditions [5,6], whereas heterogenized copper phenanthroline compounds illustrate ligand-dependent C_2_-product formation and structural stability [7,8]. Coordination design, molecular transformation and the operating catalyst environment must therefore be considered together when interpreting copper-catalyst stability.

Carbon-associated deactivation introduces a further distinction between product coordination and catalyst poisoning. On metallic copper electrodes, carbon deposition has been investigated using surface characterization and isotope-dependent Raman measurements that connect the deposited carbon to the CO_2_ feed [9]. Other work has identified electrolyte impurities as a cause of copper-electrode deactivation [10]. In heterogeneous systems, it is difficult to obtain precise atomic-level insights into the mechanisms of subsequent CO transformation; in homogeneous systems, however, EC-ESI-MS technology can be used to detect the molecular species involved in the process. In cobalt-catalyzed CO_2_ reduction, electrochemistry-coupled electrospray ionization mass spectrometry has revealed CO-bound complexes and intermediates involved in CO_2_ reduction and competing hydrogen evolution [11]. A recent floating-ground electrochemical electrospray ionization study of phenanthroline–copper catalysis further captured reactive copper-cluster intermediates and ligand transformations during CO_2_ reduction [12]. These studies have established the value of electrochemical mass spectrometry in tracking the distribution of catalyst species; however, to establish its correlation with catalyst deactivation, comparison with catalytic performance under operational conditions is required.

Pulsed electrolysis provides a means of changing both catalytic performance and the populations of retained species. Studies of heterogeneous copper electrodes have shown the influence of pulse symmetry [13], dynamic interfacial structure [14], boundary-layer transport [15], and changes in adsorbates and the local reaction environment [16,17]. A directly relevant molecular precedent is homogeneous nickel cyclam catalysis, in which pulsing improves CO selectivity through regeneration from an intermediate in the catalyst degradation pathway [18]. Pulsed operation has also promoted methane formation with N-confused copper porphyrin by tailoring the local chemical environment [19]. Consequently, an improvement under pulsing can reflect molecular regeneration, changes in intermediate formation, or interfacial effects. For the Cudpk system, the molecular question is whether alternating potentials interrupt the formation or persistence of CO-derived species before they limit subsequent CO turnover.

Assigning a reaction pathway to retained species requires atomistic structures and an energetic comparison. LASPAI combines AI-based generalized global neural network (GGNN) potential-energy evaluation with exploration of molecular configurations [20,21]. Double-ended surface walking (DESW) connects candidate endpoint structures and searches pathways and transition states [22]. Coupling DESW with GGNN energies and forces allows the competing fates of coordinated CO to be examined for Cu–dpk. The resulting relative energies must be interpreted with their reference convention: a CO-release energy difference is distinct from a verified desorption transition-state barrier, and a molecular C endpoint is distinct from a deposited carbon phase.

The mechanism governing the coordinated CO species in dissolved molecular catalyst systems during prolonged electrolysis remains unclear. Previous EC-ESI-MS studies have shown that catalyst-related intermediates can be detected, and that pulsed electrolysis has altered selectivity in other molecular systems. However, neither of these studies elucidated whether persistent carbon-coordinated metal molecules exist as carbon yield decreases, nor how alternating potentials influence their accumulation. We address this research gap by correlating Cudpk-related ions detected during CO_2_ reduction and post-electrolysis with quantified CO yields under both constant-potential and pulsed conditions. Gas-phase ion–CO reactions allow the characterization of the binding reactivity of specific ions, whilst LASPAI-based DESW/GGNN calculations compare CO release with the further transformation into copper-bound carbon species. Together, these measurements and AI-assisted calculations examine whether strong CO retention and subsequent conversion contribute to catalyst deactivation, and whether pulsed operation can limit the accumulation of the associated carbon-containing species.

## 2. Materials and Methods

### 2.1. Materials

Di(2-pyridyl) ketone (dpk, 98%) was purchased from Shanghai Aladdin Bio-Chemical Technology Co., Ltd. (Shanghai, China). Anhydrous Cu(OAc)_2_ (98%), KOH (99.9%) and acetonitrile (CH_3_CN, AR) were purchased from Shanghai McLean Bio-Chemical Co., Ltd. (Shanghai, China). KHCO_3_ (99.9%) was purchased from Shanghai Husheng Laboratory Equipment Co., Ltd. (Shanghai, China). Pure water was purchased from Wahaha Company (Nanjing, China). All reagents were used as received.

### 2.2. Catalyst Preparation Method

The catalyst preparation used Cu(OAc)_2_ (1 mmol, 0.18163 g) and di(2-pyridyl) ketone (1 mmol, approximately 0.184 g), giving a 1:1 nominal Cu:dpk feed ratio. The ligand was dissolved in 10 mL of acetonitrile, and Cu(OAc)_2_ was dissolved separately in 10 mL of water. The copper solution was added dropwise to the ligand solution, and the mixture was stirred at room temperature for 2 h. No chemical reducing agent was added. The solution was concentrated to approximately 5 mL and stored at 4 °C for 3 days. The resulting microcrystals were filtered, washed three times with cold acetonitrile and vacuum-dried at room temperature. The catalyst obtained was named Cudpk. For electrolysis, 15 mg of the isolated material was dissolved in 25 mL of 0.1 M KHCO_3_ catholyte, giving a mass concentration of 0.60 mg mL^−1^. The nominal acetate-containing formulation is [Cu(II)(dpk)(OH)]OAc, C_13_H_12_CuN_2_O_4_. Neutral dpk together with OH^−^ and OAc^−^ gives a charge-balanced Cu(II) formulation. A 6:1 molar mixture of this nominal formulation and free dpk is evaluated as a bulk-composition model. Elemental analysis combined with ICP results of the isolated material gave C 50.21%, H 4.17%, N 8.72%, O 18.69% and Cu 18.21%. The 6:1 nominal-complex/free-dpk bulk model gives C 50.26%, H 3.79%, N 9.22%, O 18.80% and Cu 17.93% (Table S1). This model provides a more accurate representation of the C/H/O ratios. The slight deviation in nitrogen (−0.50%) is within the systematic error of CHN analysis for transition-metal samples (Cu can cause incomplete combustion).

### 2.3. Electrochemical ECR Testing

CO_2_ electroreduction was performed in an H-type cell using a CHI 760F workstation (Shanghai Chenhua Instruments Co., Ltd., Shanghai, China) and a three-electrode arrangement. A 3 mm diameter glassy carbon electrode (Wuhan Gaoshiruilian Technology Co., Ltd., Wuhan, China) (geometric area approximately 0.071 cm^2^) was the working electrode, Ag/AgCl in saturated KCl (Wuhan Gaoshiruilian Technology Co., Ltd., Wuhan, China) was the reference, and a Pt wire (Wuhan Gaoshiruilian Technology Co., Ltd., Wuhan, China) was the counter electrode. The glassy carbon electrode was polished and rinsed before use. The catalyst was dissolved in the catholyte; no catalyst coating or immobilization step was used. The catholyte and anolyte each consisted of 25 mL of 0.1 M KHCO_3_, and the chambers were separated by a Nafion-117 membrane. The catholyte contained 15 mg of dissolved microcrystals (0.60 mg mL^−1^). Prior to measurement, the cathode cell was purged with high-purity CO_2_ for 30 min, and the CO_2_-saturated electrolyte pH was 6.8. CO_2_ (99.99%) was supplied at 25 mL min^−1^, and the outlet flow was monitored using a soap-bubble flow meter. Experiments were conducted at ambient temperature and pressure without iR compensation. All potentials vs. the Ag/AgCl electrode were converted to the potentials vs. RHE based on the following equation:

E (vs. RHE) = E (vs. Ag/AgCl) + 0.197 + 0.059 × pH

Pulsed electrolysis alternated a reduction potential of −0.53 V and an oxidation potential of +0.53 V vs. RHE, each held for 180 s. For a complete reduction/oxidation pair, the period is 360 s, the frequency is 2.78 × 10^−3^ Hz and the reduction duty cycle is 50%. The short sequence shown in Figure S1 contains five reduction segments and four oxidation segments, begins and ends with reduction. A 16 h record corresponds to 160 complete 360 s pairs. The complete pulse parameters are given in Table S2.

### 2.4. EC-ESI-MS Experimental Method

EC-ESI-MS measurements used an LTQ XL linear ion trap mass spectrometer (Thermo Fisher Scientific, Waltham, MA, USA) with an ESI source. A ChenHua workstation controlled the potential in the custom electrochemical flow cell. Ar or CO_2_ overpressure (0.2–0.4 bar) transferred solution from the cell toward the MS inlet. Typical source settings were a capillary temperature of 275 °C, spray voltage of 4–5 kV, capillary voltage of 0 V and tube-lens voltage of 10–40 V. Acetonitrile was introduced at the MS inlet interface for ionization; it was not a component of the bulk electrolysis formulation. Consequently, [Cu(dpk)(CH_3_CN)]^+^ denotes an MS adduct. Source-related Cu(II)-to-Cu(I) reduction is documented in ESI, including aqueous acetonitrile and gas-phase solvent–complex charge transfer [23,24], so the detected ion charge and its formal metal-valence assignment are distinguished from the oxidation state of the prepared catalyst. Mass-selected collision-induced dissociation (CID) used an isolation width of 1 *m*/*z*. The values labeled in volts in Figures S2, S7 and S8 are collision voltages.

### 2.5. Gas-Phase Ion-Molecule Reaction Method in Ion Trap

Gas-phase ion-molecule reactions were measured using a modified ESI-LTQ XL linear ion trap. The [Cu(dpk)(CH_3_CN)]^+^ ion at *m*/*z* = 288 was mass-selected, and CO was introduced at an effective pressure of approximately 1 × 10^−5^ Torr. Catalyst solution was introduced through ESI at 5 μL min^−1^. The *m*/*z* = 316 CO adduct was the only detected reaction product in this experiment. The natural logarithm of the normalized residual parent abundance was fitted against delay using a free intercept and weights of 1/u_i_^2^, where u_i_ is the supplied pointwise uncertainty. Normalization, data and rate conversion are given in Supplementary Section S1. The measured rate characterizes the isolated gas-phase ion under trap conditions.

### 2.6. Product Quantification

The outlet stream was sampled online by GC (Zhejiang Fuli Analytical Instrument Co., Ltd., Wenling, China). Separation used TDX-01 (Zhejiang Fuli Analytical Instrument Co., Ltd., Wenling, China), HP-PLOT Al_2_O_3_ S (Agilent Technologies, Santa Clara, CA, USA) and Porapak Q columns (Agilent Technologies, Santa Clara, CA, USA) with high-purity Ar carrier gas. FID channels monitored C_1_ and C_2_+ products, and TCD monitored H_2_ and other permanent gases. CO quantification used a three-point calibration at 0, 100 and 50,000 ppm (Figure S5). The instrument-reported detection limit for CO is 5–100,000 ppm. Detection limits were as low as 0.1 ppm for CH_4_ and C_2+_ products. The reported calibration R^2^ rounds to 1 at the displayed precision. Product amounts were calculated from GC quantification and outlet flow, and FE was calculated from product amounts and cathodic charge. The reported FE data are based on three independent experiments. For gas-phase products, the FE was calculated using the following equation:

FE_product_ = (C_product_ × V × N × F)/Q_c_ × 100%

where C_product_ is the GC-measured concentration (mol/L),

V is the volume of the outflow;

N = 2 is the number of electrons transferred for CO_2_-to-CO conversion;

F = 96,485 C/mol is the Faraday constant;

Q_c_ represents the total charge of the cathodic period.

Representative flow-based production rates were approximately 0.454 μmol min^−1^ for CO and 0.141 μmol min^−1^ for H_2_ (27.24 and 8.47 μmol h^−1^, respectively), using an outlet flow of 25 mL min^−1^ and a molar gas volume of 24.45 L mol^−1^ (298 K and 1 atm). These representative rates are reported separately from the optimum FE values. Liquid products were examined by ^1^H NMR using a Bruker 400 MHz instrument (Billerica, MA, USA). After electrolysis, a known amount of DMSO was added as the quantitative internal standard. D_2_O was used for field locking. No liquid products were detected under these analytical conditions.

### 2.7. LASPAI-Based AI Calculations

To obtain suitable endpoint geometries for reaction pathway searches, the initial- and final-state structures were constructed and optimized using the Generalized Global Neural Network Potential (GGNN) [21]. Reaction pathways were subsequently explored using the double-ended surface walking (DESW) [25] method on the LASPAI platform (www.laspai.com) [22], with energies and forces evaluated using GGNN.

## 3. Results

### 3.1. Structural Analysis

**Figure 1:**
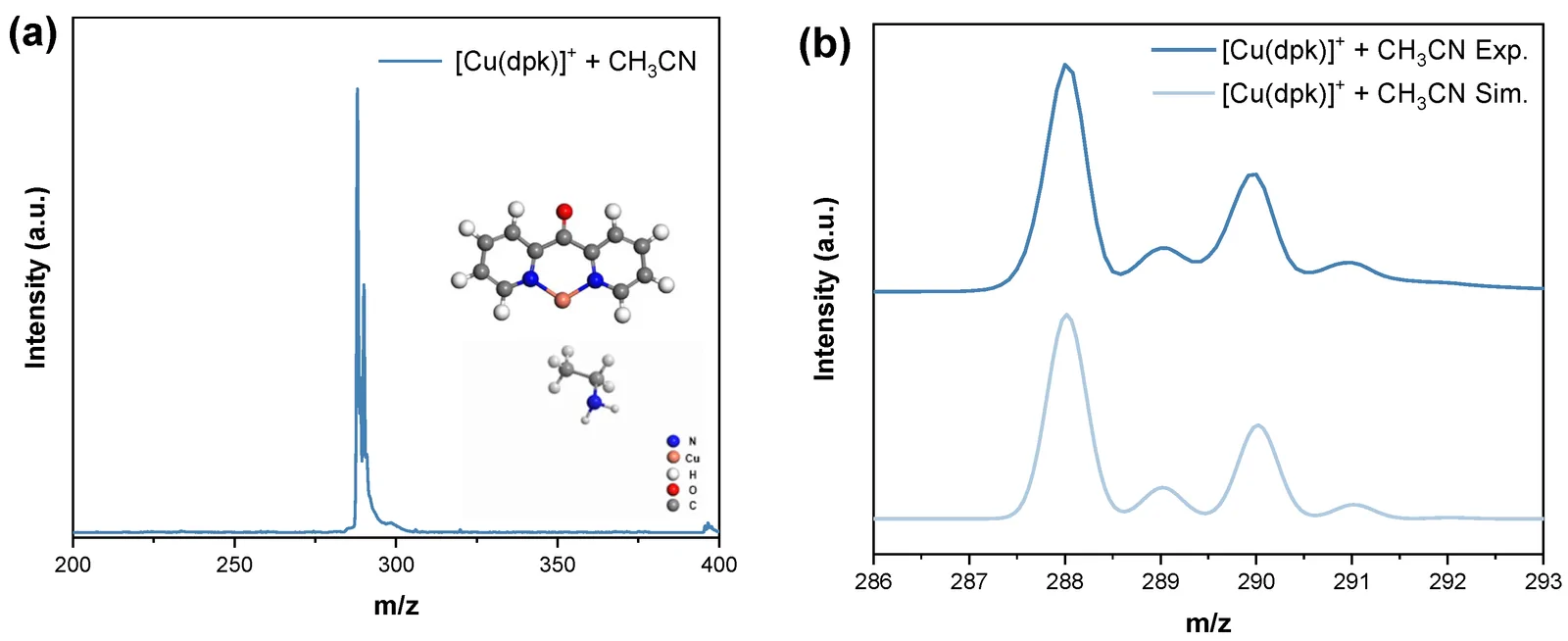
(**a**) Positive-mode ESI-MS spectrum of the Cu–dpk catalyst solution with acetonitrile introduced for ionization; the inset shows the proposed adduct. (**b**) Experimental and simulated isotope envelopes for [Cu(dpk)(CH_3_CN)]^+^.

The prepared material is formulated as a Cu(II)-based Cu–dpk precursor: Cu(OAc)_2_ was used without an added chemical reducing agent, and the proposed neutral acetate/hydroxo composition is charge-balanced for Cu(II). Elemental analysis supports consideration of that overall formulation. Positive-mode ESI-MS instead detects the prominent *m*/*z* = 288 ion consistent with [Cu(dpk)(CH_3_CN)]^+^, with agreement between measured and simulated copper isotope envelopes (Figure 1). CID yields *m*/*z* = 247, assigned as [Cu(dpk)]^+^. If dpk and CH_3_CN are neutral ligands, these monocations have a formal Cu(I) assignment; the observed +1 is also the total charge of the molecular ion. ESI-related copper reduction provides a documented explanation for obtaining such ions from a Cu(II) starting material [23,24].

CID of the *m*/*z* = 288 precursor at the displayed settings of 0–12 V generated a principal fragment at *m*/*z* = 247 (Figure S2). The 41 Da difference is consistent with loss of acetonitrile to yield [Cu(dpk)]^+^. No prominent ligand-backbone or metal–ligand cleavage fragments were apparent within the displayed mass range and collision settings. These observations support retention of the Cu–dpk core during this gas-phase fragmentation step; they do not establish bulk purity or persistence of the molecular structure at all stages of electrolysis.

### 3.2. Electrochemical Characterization

**Figure 2:**
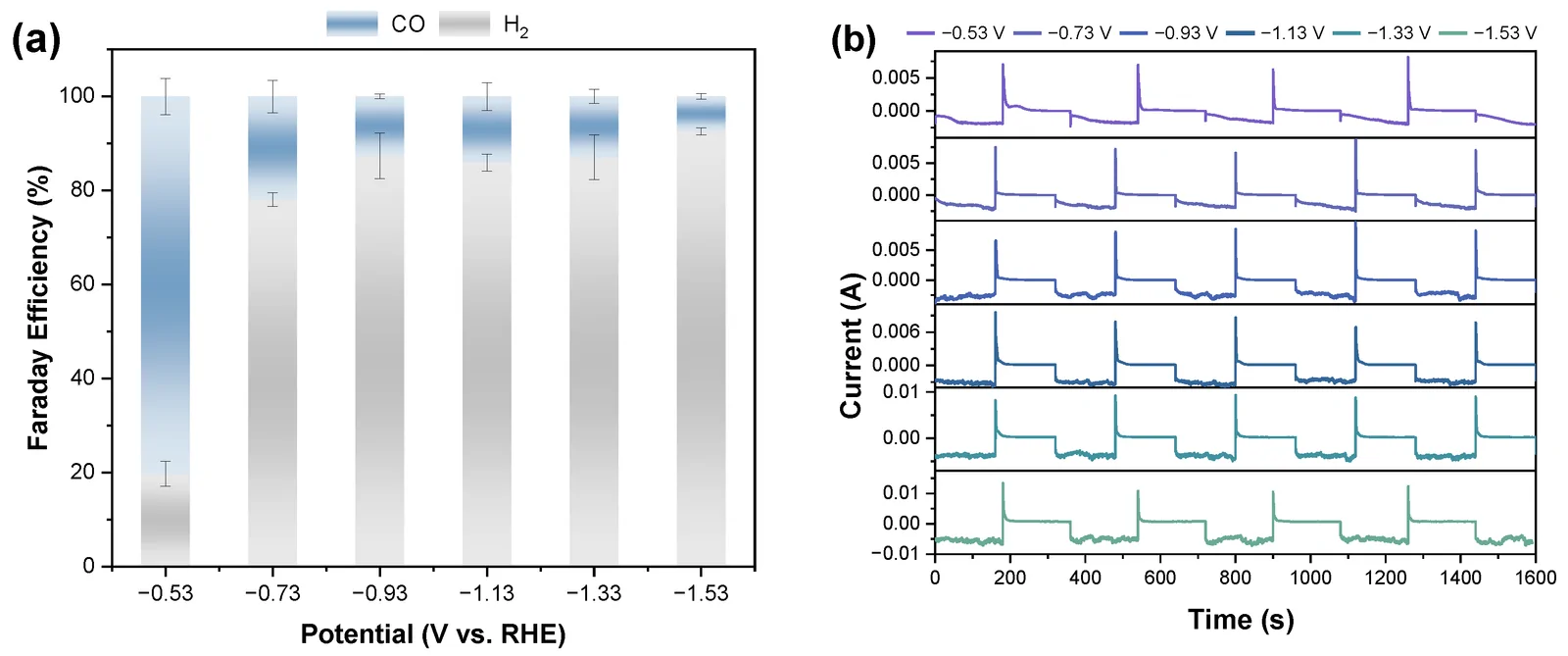
(**a**) Faradaic efficiencies for CO and H_2_ at different reduction potentials. (**b**) Current–time responses during alternating-potential electrolysis at the indicated reduction potentials vs. RHE.

The CV (Figure S3) and LSV (Figure S4) show that the cathodic current is higher in CO_2_-saturated electrolyte than under Ar conditions. The quantitative detection of CO during the CO_2_-fed electrolysis, coupled with the absence of carbon-containing products in the Ar electrolysis control group, supports the attribution of this additional response to CO_2_-dependent CO generation. Under the stated analytical conditions, no carbon-containing products were detected in the Ar electrolysis control group. Consequently, CO_2_ was identified as the effective carbon source for the detected CO, and no contribution from carbon products derived from dpk or acetate was detected in this control group. No liquid products were detected by ^1^H NMR following electrolysis. CO was the only detected carbon-containing gaseous product across the investigated reduction potentials, with H_2_ as the competing gaseous product (Figure 2a). An optimum CO Faradaic efficiency (FE) of 79.33% was achieved at a pulse potential of −0.53 V vs. RHE. Electrochemical product measurements were carried out in three independent experiments.

After approximately 11,000 s of electrolysis at a constant potential, the measured gas composition was almost entirely H_2_. At the same time point, the CO FE reported in pulse operation mode remained at 77.91%, and the duration of the pulse current curve was extended to approximately 16 h (Figure S6).

Following constant-potential electrolysis, an additional m/z = 260 ion appeared in the mass spectrum of the solution; its composition was identified as [Cu(dpk)CH]^+^, whilst CID produced a signal at *m*/*z* = 247. Following 16 h of pulsed electrolysis, the acetonitrile-assisted mass spectrometry spectrum was dominated by the Cudpk adduct at *m*/*z* = 288, with no discernible signal at *m*/*z* = 260 (Figure S7). This comparative end-point analysis indicates that the detected CH-related species had been reduced. Following pulsed electrolysis, the electrode surface appeared clean upon visual inspection. The electrode was rinsed with water and then subjected to ultrasonic treatment in ethanol to prepare a dispersion. No obvious metallic or carbon-containing substances were observed. It is worth noting that, under the applied pulse potential, CO release remains the predominant reaction pathway observed; however, during each reduction cycle, a small fraction of residual CO (insufficient to be quantified by the Faraday method) may form persistent copper-bound carbonaceous species. Oxidation pulses may limit the accumulation of these species, whilst sustained reduction may lead to the accumulation of trace amounts of these species, ultimately resulting in the blocking of catalytic sites.

### 3.3. Mechanism Analysis

**Figure 3:**
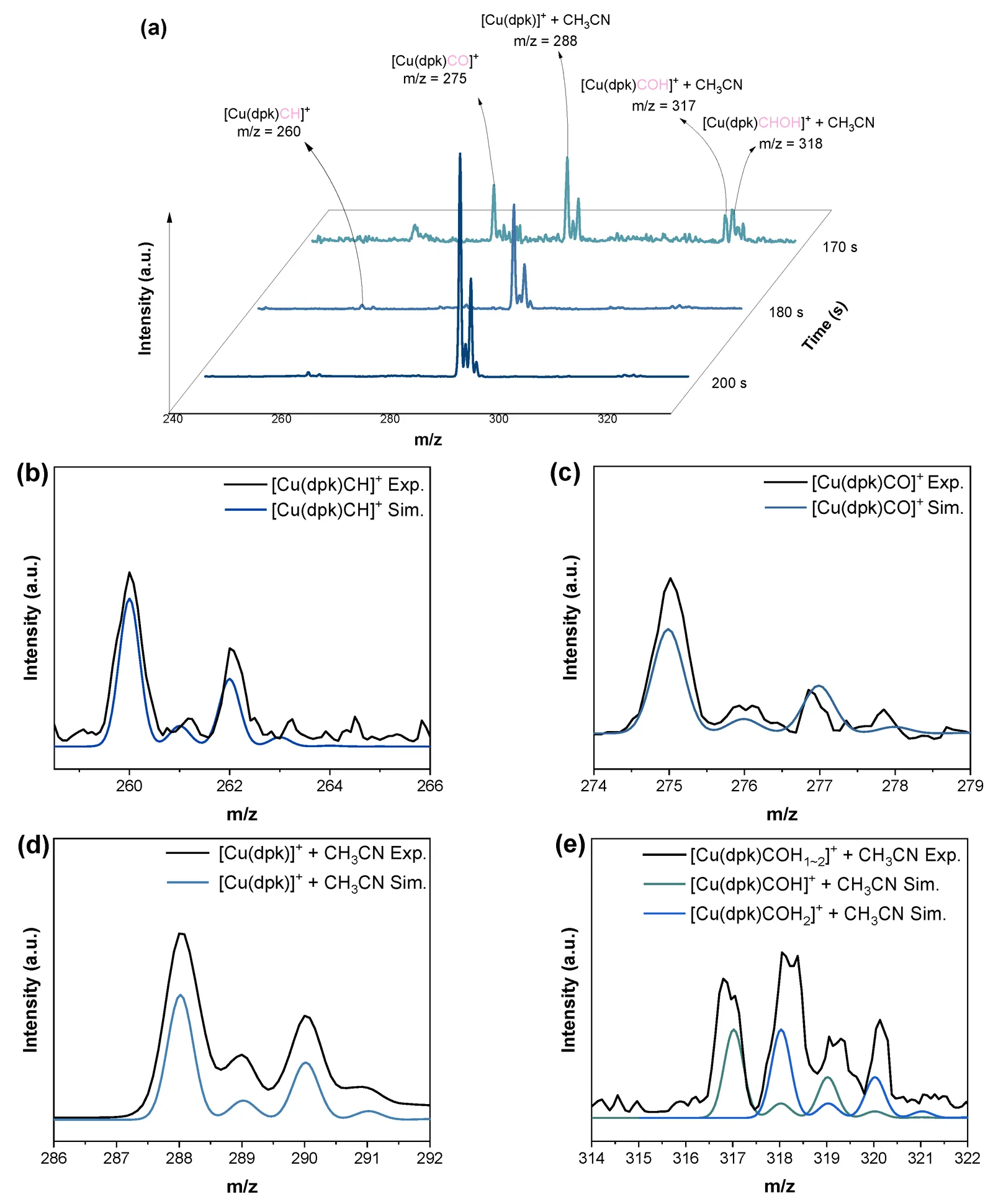
(**a**) EC-ESI-MS spectra at electrolysis times of 170, 180 and 200 s under reducing current conditions. For the EC-ESI-MS measurements, a constant reducing potential was applied in i–t mode because the sampling period was short and the cathodic reduction products needed to be transferred to the mass spectrometer for detection. (**b**–**e**) Experimental and simulated isotope envelopes for the proposed [Cu(dpk)CH]^+^, [Cu(dpk)CO]^+^, [Cu(dpk)(CH_3_CN)]^+^ and [Cu(dpk)COH_1~2_(CH_3_CN)]^+^ compositions. Composition assignments are supported by CID in Figure S8.

EC-ESI-MS detected Cudpk-associated ions at *m*/*z* = 275, 260, 317 and 318, in addition to the acetonitrile adduct at *m*/*z* = 288 (Figure 3). Their isotope patterns and CID behavior support composition-level assignments to [Cu(dpk)CO]^+^ (*m*/*z* = 275), [Cu(dpk)CH]^+^ (*m*/*z* = 260), [Cu(dpk)COH(CH_3_CN)]^+^ (*m*/*z* = 317) and [Cu(dpk)CHOH(CH_3_CN)]^+^ (*m*/*z* = 318), respectively (Figure S8). These observations suggest that a branch point may exist after the formation of a Cu-associated CO species. CO release would contribute to the measured gaseous product, whereas CO retention may allow further proton-coupled transformation to COH- and CHOH-associated species. The oxygen-free CH-associated composition is also consistent with a possible continuation involving C–O bond cleavage and retention of carbon at the Cudpk core.

The corresponding carbon-associated signals were not observed in the Ar-saturated KOH EC-ESI-MS control, in which acetonitrile and water adducts predominated (Figure S9). This comparison supports their association with the CO_2_-fed electrolysis conditions. In separate post-electrolysis solution samples, the *m*/*z* = 260 CH-associated ion was detected after constant-potential operation but was not obvious after approximately 16 h of pulsed operation. Together with the different CO/H_2_ product distributions, this comparison suggests reduced persistence or formation of CH-associated species under pulsed operation, but does not establish oxidative removal as the sole mechanism. The resulting mechanistic question is whether retained Cu-bound CO can energetically compete between release as CO and further conversion into a persistent carbon state. The LASPAI-assisted pathway analysis below examines these alternative fates of bound CO.

The LASPAI profile below provides a candidate chemical connection from retained CO through COH toward a Cu-bound C state.

**Figure 4:**
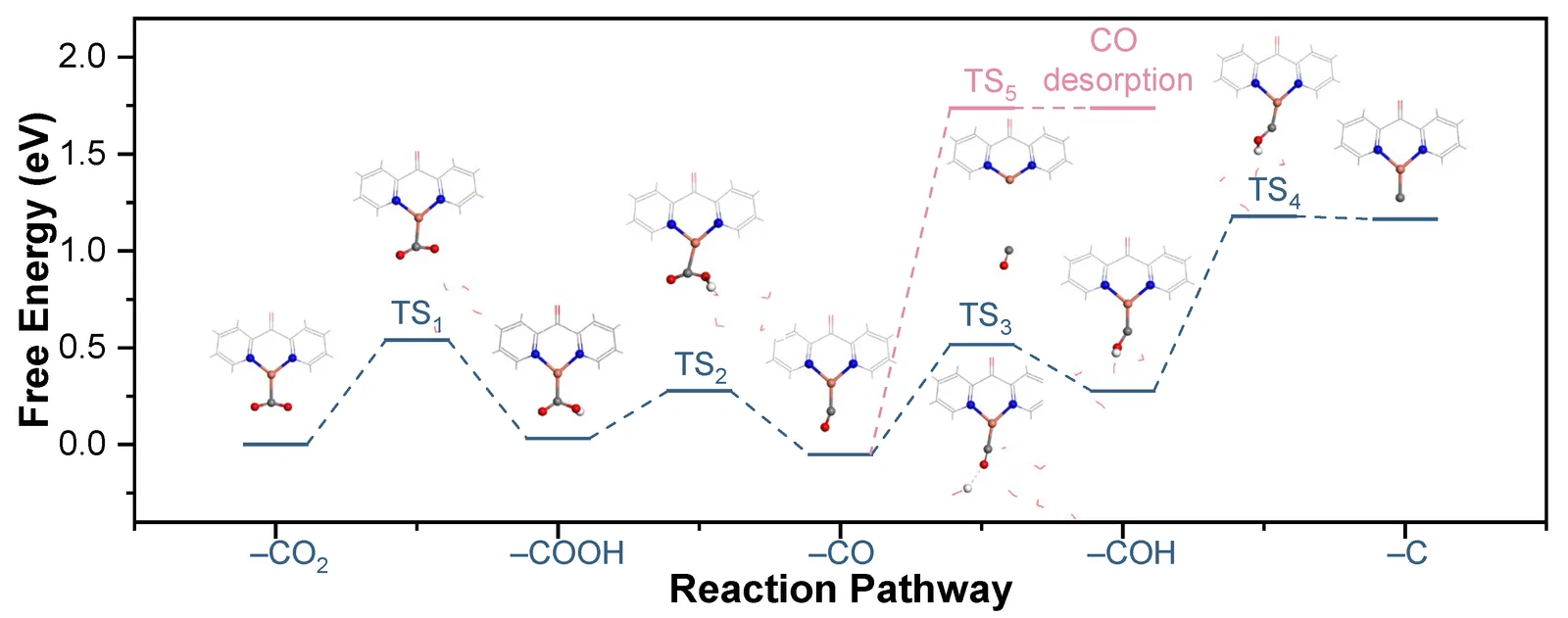
AI-assisted LASPAI relative-energy profile for Cu-bound CO_2_ → COOH → CO → COH → C obtained with DESW pathway searches and GGNN free-energy evaluations. TS_1_–TS_5_ are the candidate transition-state configurations labeled in the reported profile.

The LASPAI-assisted DESW/GGNN energy profile provides an atomistic framework for the competing fates of Cu-bound CO inferred from the EC-ESI-MS results (Figure 4). On the energy scale, the CO-release branch lies approximately 1.73 eV above the bound CO state, supporting strong CO retention in the Cudpk model. This energy difference is not an independently determined desorption activation barrier. Along the competing pathway from bound CO through COH to Cu-bound C, the local energy differences relative to TS_3_ and TS_4_ are approximately 0.57 and 0.84 eV, respectively. Thus, the calculated profile supports the energetic plausibility of further conversion while CO remains coordinated, although the Cu-bound C endpoint represents a molecular model state rather than an identified carbon deposit.

The experimental product distribution provides important supporting evidence for this picture. CO is the principal quantifiable carbon product, with a reported Faradaic efficiency of 79.33% at the optimum measurement point. Therefore, under the actual solvated and potential-controlled conditions, CO release may be more thermodynamically favorable than the model energy profile alone suggests, while the calculated values support the possibility of conversion from CO toward carbon-containing species. Even if, in each reduction cycle, only a small amount of residual CO (insufficient for Faradaic quantification) follows carbon-related conversion pathways, its persistent presence may gradually reduce the number of available catalytic sites during constant-potential operation. To examine another aspect of the proposed Cu–CO interaction, we next measured the gas-phase reaction of CO with mass-selected [Cu(dpk)(CH_3_CN)]^+^. This gas-phase ion-molecule experiment quantifies the rate of CO association with the isolated Cudpk ion, complementing the LASPAI-derived energetic evidence for strong CO retention in the model.

**Figure 5:**
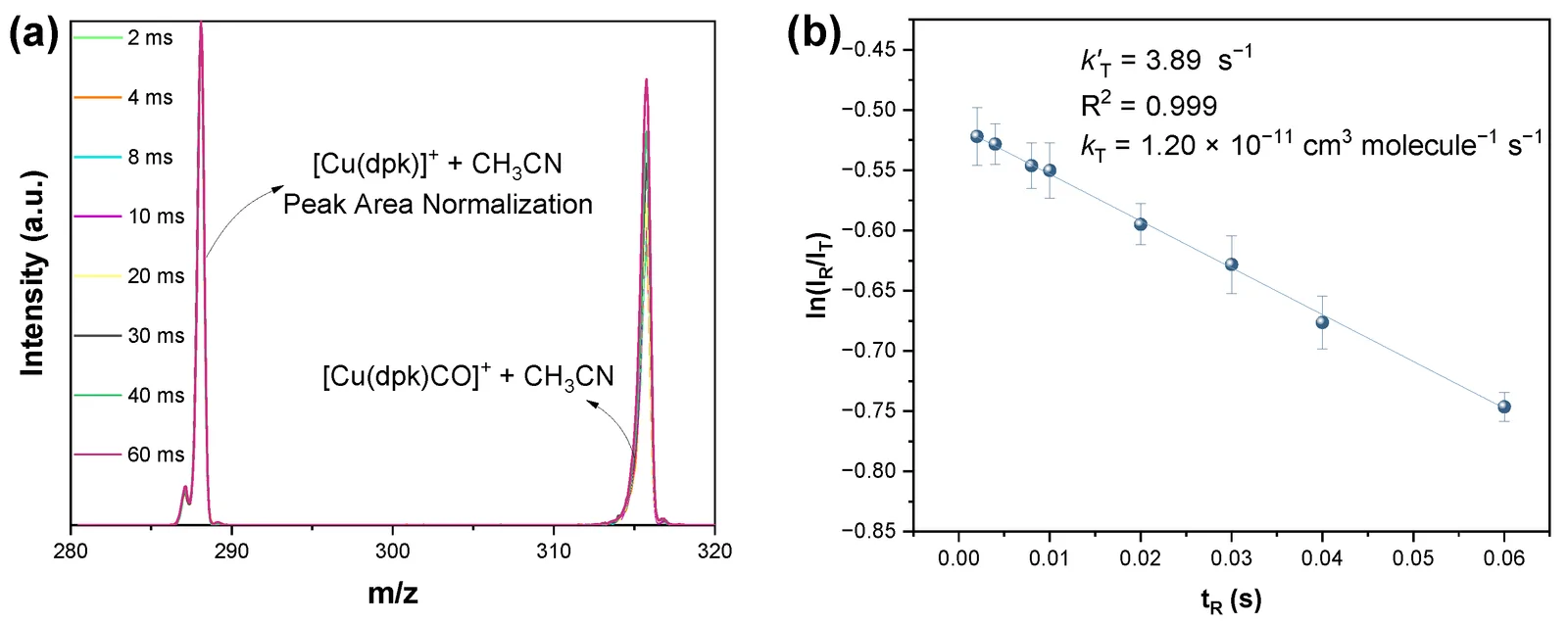
Gas-phase reaction of mass-selected [Cu(dpk)(CH_3_CN)]^+^ with CO. (**a**) Normalized spectra at eight delays of 2–60 ms. (**b**) Kinetic fitting for rate-constant extraction of the ion-molecule reaction. The error bars are based on three independent experiments.

The mass-selected [Cu(dpk)(CH_3_CN)]^+^ precursor at *m*/*z* = 288 reacted with CO to produce *m*/*z* = 316, consistent with addition of one CO molecule (Figure 5). Eight delays between 2 and 60 ms were measured. An inverse-variance-weighted linear fit of ln(I_R_/I_T_) with a free intercept gave *k*′_T_ = 3.89 s^−1^ and weighted R^2^ = 0.99912. The CO adduct was the only detected product in this gas-phase reaction, so no competing-product branching correction was applied. At approximately 1 × 10^−5^ Torr and 298 K, n_CO_ = 3.24 × 10^11^ molecules cm^−3^ and *k*_T_ = 1.20 × 10^−11^ cm^3^ molecule^−1^ s^−1^. The fit data and regression uncertainty are given in Supplementary Section S1. These results establish kinetic accessibility of CO coordination to the isolated ion. The observed CO-adduct formation demonstrates that the isolated Cudpk ion can coordinate CO; together with the high energetic cost of CO release predicted by LASPAI, it supports strong CO binding at the Cudpk center.

To assess whether the strong CO retention suggested by gas-phase ion-molecule experiments and LASPAI calculations is also reflected in the electrochemical behavior of the Cudpk system, CO-saturated voltammetry was performed using a polished bare glassy carbon electrode in the dissolved catalyst solution (Figure S10). The solution was purged with CO for 30 min, the CO flow was then stopped, and repeated voltammograms were recorded at 100 mV s^−1^ in 0.1 M KHCO_3_ over the corrected window of −1.825 to +3.165 V vs. RHE. An Ar-saturated catalyst CV recorded under the same conditions supplied the background. Current was subtracted pointwise at matched potential and scan direction. The anodic CO-associated response diminished over repeated scans and approached overlap after approximately the eleventh scan. The adsorbed CO remains on the catalyst center for a relatively long time; consequently, it is difficult to desorb it completely within a single oxidation scan. This conclusion is highly consistent with the in situ EC-ESI-MS observations of the deep hydrogenation intermediates, further corroborating the mechanistic explanation of the aforementioned deactivation mechanism.

## 4. Discussion

Previous molecular CO_2_-reduction studies illustrate how relatively labile CO binding can favor product release: weak binding limits further CO reduction in immobilized cobalt protoporphyrin [26], while a cobalt porphyrin model predicts favorable CO dissociation and catalyst regeneration [27]. Cudpk provides a complementary system for examining the consequences of strong CO retention. The AI-assisted LASPAI pathway analysis identifies a substantial energetic cost for CO release and an energetically plausible continuation toward a copper-bound carbon state. Together with the persistence of CH-associated ions after constant-potential electrolysis, these findings connect strong CO retention to a candidate carbon-associated poisoning pathway. Although CO remains the dominant quantified carbon product, a small, unquantified fraction of retained intermediates could progressively occupy available Cu sites and impair subsequent turnover. The mechanistic purpose of pulsed operation is therefore to interrupt this accumulation and recover sites for continued CO production.

This proposed poisoning pathway differs from the principal emphasis of representative pulsed-electrolysis studies on heterogeneous copper. Arán-Ais and co-workers associated improved selectivity with pulse-generated morphological motifs and Cu(I) species [28], whereas Timoshenko and co-workers used potential pulses to control structural and chemical transformations of copper catalysts [29]. In Cudpk, potential modulation is examined specifically as a means of suppressing the persistence of carbon-associated species arising from strongly retained CO. The oxidative interval interrupts continuous reduction and may promote removal of these species, allowing subsequent cathodic intervals to resume CO-forming turnover. The reduced persistence of the CH-associated ion after prolonged pulsed operation is consistent with this interpretation. Following the pulse experiment, the CH-correlated signal weakened whilst CO production persisted, supporting the notion that the accumulation of carbon-correlated species was suppressed; however, this does not yet prove that oxidative clearance is the sole recovery mechanism. Consequently, the proposed carbon-correlated poisoning pathway is a mechanistic explanation supported by both experimental and computational evidence. Combining AI-guided identification of these poisoning pathways with pulse protocols tailored to site recovery offers a route for developing durable molecular catalysts that sustain CO production despite strong CO binding.

## Data Availability

The original contributions presented in this study are included in the article and Supplementary Material. Further inquiries can be directed to the corresponding author.

## Supplementary Materials

The following supporting information can be downloaded at https://www.mdpi.com/article/10.3390/ma19214500/s1, Figure S1: Programmed short pulse waveform. Five reduction segments at −0.53 V vs. RHE and four oxidation segments at +0.53 V vs. RHE, each 180 s, give a total duration of 1620 s. The sequence begins and ends with reduction. Long-term operation repeats complete reduction/oxidation pairs; Figure S2: CID of [Cu(dpk)(CH_3_CN)]^+^ at *m*/*z* = 288 at the displayed settings: (a) 0 V, (b) 4 V, (c) 8 V and (d) 12 V. The *m*/*z* = 247 fragment is consistent with a nominal 41 Da loss of CH_3_CN; Figure S3: CV of the catalyst solution under Ar (green) and CO_2_ (blue); Figure S4: LSV of the catalyst solution under Ar (green) and CO_2_ (blue); Figure S5: CO GC calibration (0, 100 and 50,000 ppm). Fit: A (fA·s) = 7896.41828cCO (ppm) + 18615.43339; R^2^ ≈ 1 at displayed precision. The inset shows 0–140 ppm and the same three-point fit; Figure S6: Current–time traces during (a) constant-potential electrolysis at −0.53 V vs. RHE and (b) pulsed electrolysis with E_red_ = −0.53 V and E_ox_ = +0.53 V vs. RHE, each held for 180 s; Figure S7: (a) Solution MS after constant-potential electrolysis. (b) Solution MS after approximately 16 h of pulsed electrolysis. (c) CID of the mass-selected *m*/*z* = 260 ion, with a fragment at *m*/*z* = 247; Figure S8: CID spectra supporting the proposed compositions of (a) [Cu(dpk)CH]^+^, *m*/*z* = 260, at 18 V; (b) [Cu(dpk)CO]^+^, *m*/*z* = 275, at 3 V; (c) [Cu(dpk)COH(CH_3_CN)]^+^, *m*/*z* = 317, at 8 V; and (d) [Cu(dpk)CHOH(CH_3_CN)]^+^, *m*/*z* = 318, at 5 V; Figure S9: EC-ESI-MS of the catalyst solution in Ar-saturated KOH electrolyte; Figure S10: Ar-background-subtracted anodic current during repeated CO-saturated voltammetric scans in dissolved Cudpk catalyst solution. CO and Ar currents were matched at the same potential and scan direction and subtracted pointwise. The displayed segment is 1.0–1.7 V vs. RHE within the full scan window of −1.825 to +3.165 V vs. RHE. Table S1: The elemental composition determined experimentally and the 6:1 molar mixture composition derived theoretically; Table S2: Pulse parameters.
