# Supplementary material for: LASPAI-Assisted Mechanistic Investigation of Carbon Poisoning in Molecular Copper-Catalyzed CO2 Reduction
Source: Materials (Basel). 2026 Oct 26;19(21):4500. doi: 10.3390/ma19214500 (PMC13764414; doi:10.3390/ma19214500)
Supplement: Supplementary file 1 [file materials-19-04500-s001.zip › materials-4601951-supplementary.pdf]

Supplementary Information

LASPAI-Assisted Mechanistic Investigation of Carbon Poisoning in Molecular Copper-Catalyzed CO_2 Reduction

Shanshan Lin [1,2,*], Guangjing Feng [2] and Xiaoyan Sun [1,2]

[1] State Key Laboratory of Photoelectric Conversion and Utilization of Solar Energy, Qingdao New Energy Shandong Laboratory, Qingdao Institute of Bioenergy and Bioprocess Technology, Chinese Academy of Sciences, Qingdao 266101, China; sunxy@qibebt.ac.cn

[2] University of Chinese Academy of Sciences, Beijing 100049, China; fengguangjing23@mails.ucas.ac.cn

* Correspondence: linss@qibebt.ac.cn

S1. Gas-phase ion-molecule reaction method in ion trap

A modified ESI-LTQ XL instrument was used for gas-phase reactions. A diluted Cu–dpk catalyst solution was introduced through ESI at 5 μL min^{-1}. Acetonitrile-assisted ionization provided the $[Cu(dpk)(CH_3CN)]^+$ precursor. The m/z = 288 ion was isolated in the linear ion trap, CO was introduced at an effective pressure of approximately 1×10^{-5} Torr, and the resulting spectrum was recorded after a defined delay between isolation and ejection. The reaction rate can be calculated by determining the concentrations of the reactants at different reaction times; the formula for the rate constant of a quasi-first-order reaction is as follows:

$$ln\frac{I_\mathrm{R}}{I_\mathrm{T}} = -k'_\mathrm{T}t_\mathrm{R} \qquad \text{S1}$$

$$ln\frac{I_\mathrm{R}}{I_\mathrm{T}} = -k_\mathrm{T}\frac{P_\mathrm{effective}}{k_\mathrm{T}}t_\mathrm{R} \qquad \text{S2}$$

$$P_\mathrm{effective} = \frac{NkT}{V} \qquad \text{S3}$$

$$k_\mathrm{T} = \frac{k'_\mathrm{T}V}{N} \qquad \text{S4}$$

The terms in the formula are, respectively:

I_R—Peak area of cluster A after the reaction;

I_T—The sum of the peak areas of cluster A and all products after the reaction;

k'_T—Quasi-first-order reaction rate constant (s^{-1});

k_T—Bimolecular rate coefficient (cm^3 s^{-1} $molecule^{-1}$);

$P_{effective}$—The pressure of the reaction gas introduced into the ion trap;

k—The Boltzmann constant, 1.38×10^{-23} J/K;

T—Reaction temperature, 298 K;

t_R—Reaction time;

V—Ion trap volume, 1.30×10^5 $mm^{3;}$

N—Number of reactant molecules.

However, due to the presence of impurity gases such as water and solvent molecules in the linear ion trap, the observed reaction rate (kT) represents the total adsorption rate of various gas molecules onto cluster ions. Based on the characteristics of parallel reactions—namely, that when all parallel reactions are of the same order, and the initial concentration of the products is zero, the ratio of the concentrations of the various products at any point in time is equal to the ratio of the reaction rate constants—we can therefore calculate the reaction rate constant (k_{gas}) for the target reaction using Equation 5:

$$\frac{k_\mathrm{gas}}{k_\mathrm{T}} = \frac{I_\mathrm{gas}}{I_\mathrm{T}} \qquad \text{S5}$$

The terms in the formula are, respectively:

I_{gas}—Peak area of cluster ions adsorbing the reaction gas following the reaction;

k_{gas}—Quasi-first-order reaction rate for reactant adsorption.

The pointwise vertical error bars represent $\pm u_i$ in $\ln(I_r/I_t)$; the u_i values mean 0.01975. The weighted fit gives an intercept of −0.514248 and a slope of −3.888554 s^{-1}; hence k′CO = k′obs = 3.89 s^{-1}, with a weighted R^2 of 0.99912. The slope standard error is 0.0470 s^{-1}, calculated from the weighted regression with residual-variance scaling and six residual degrees of freedom; the corresponding model-based 95% confidence interval for k′CO is 3.77–4.00 s^{-1}. At Peffective ≈ 1 × 10^{-5} Torr (1.333 × 10^{-3} Pa) and T ≈ 298 K, nCO ≈ 3.240 × 10^{11} molecules cm^{-3}, giving k_2 ≈ 1.20 × 10^{-11} cm^3 $molecule^{-1}$ s^{-1}. Propagation of the fitting standard error alone gives an SE of 1.45 × 10^{-13} cm^3 $molecule^{-1}$ s^{-1} for k_2, assuming nCO is fixed. These fitting uncertainties describe this representative curve. Pressure calibration and other systematic uncertainties are not included.

S2. Supplementary Figures

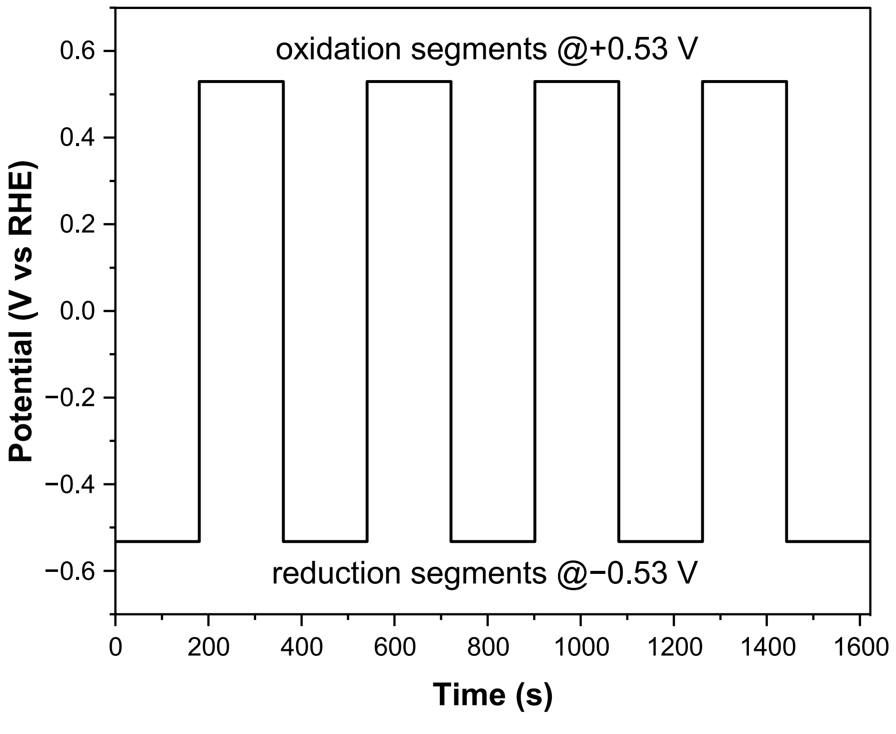

Figure S1. Programmed short pulse waveform. Five reduction segments at −0.53 V vs. RHE and four oxidation segments at +0.53 V vs. RHE, each 180 s, give a total duration of 1620 s. The sequence begins and ends with reduction. Long-term operation repeats complete reduction/oxidation pairs.

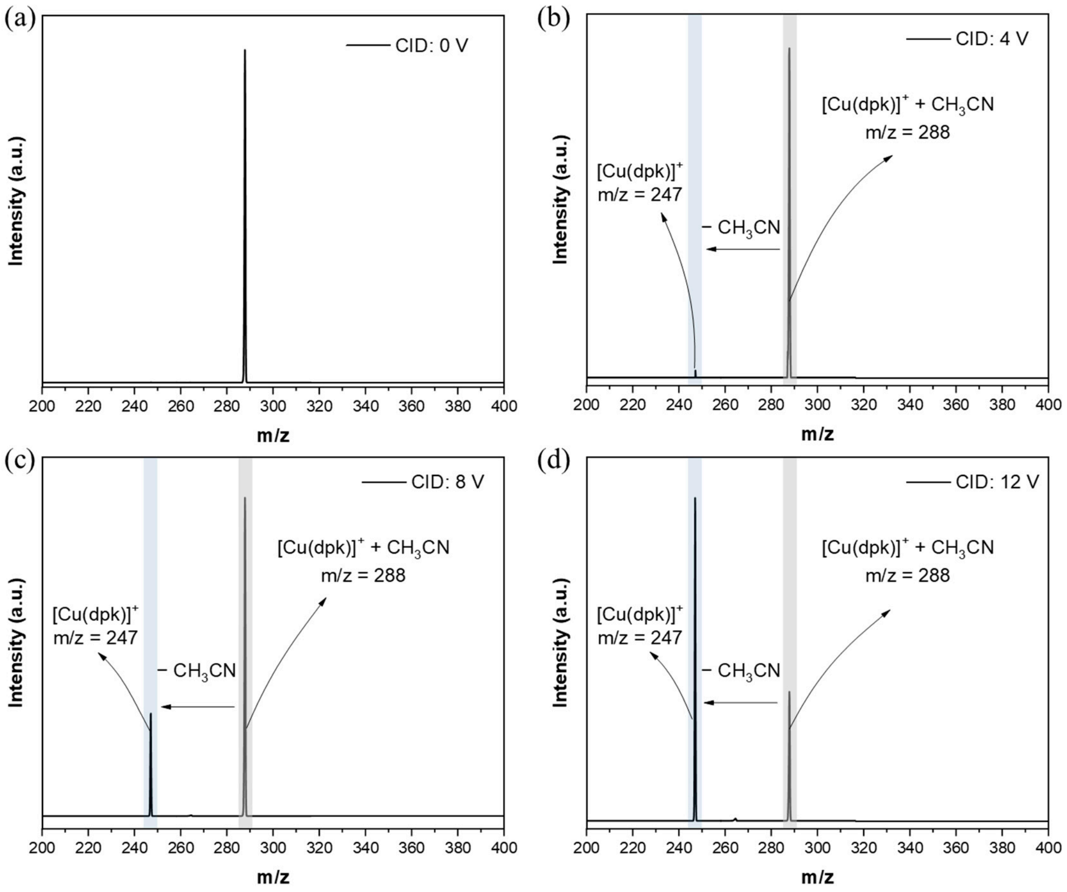

Figure S2. CID of $[Cu(dpk)(CH_3CN)]^+$ at m/z = 288 at the displayed settings: (a) 0 V, (b) 4 V, (c) 8 V and (d) 12 V. The m/z = 247 fragment is consistent with a nominal 41 Da loss of CH_3CN.

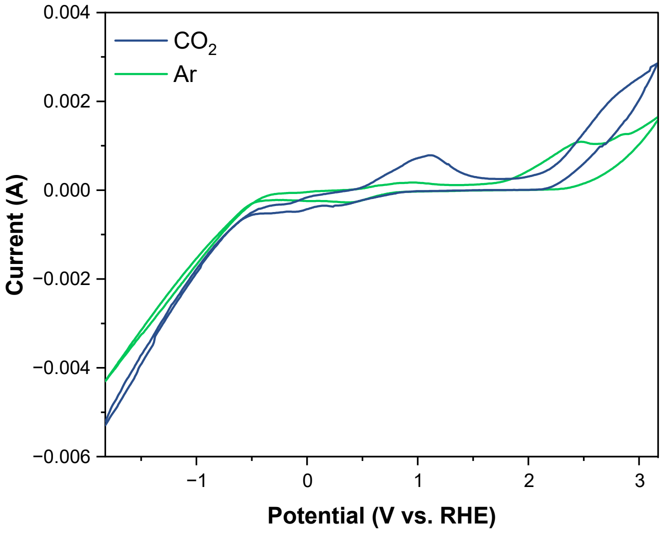

Figure S3. CV of the catalyst solution under Ar (green) and CO_2 (blue).

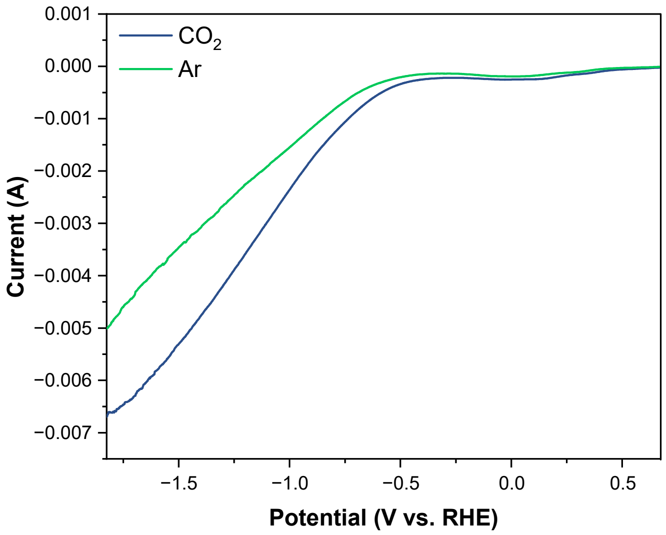

Figure S4. LSV of the catalyst solution under Ar (green) and CO_2 (blue).

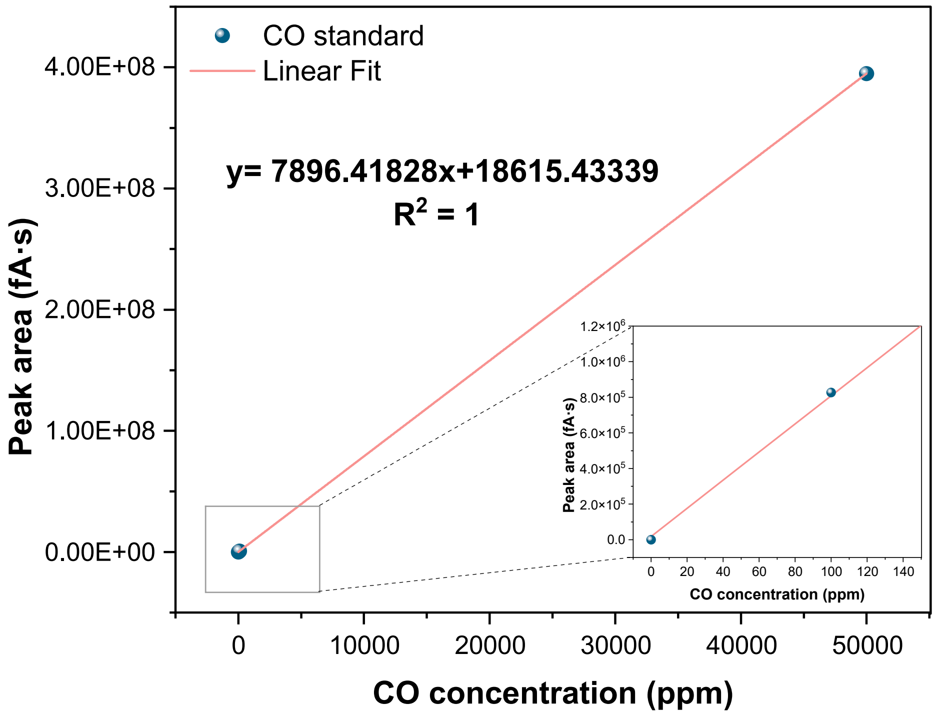

Figure S5. CO GC calibration (0, 100 and 50,000 ppm). Fit: A (fA·s) = 7896.41828c_{CO} (ppm) + 18615.43339; $R^2 \approx 1$ at displayed precision. The inset shows 0–140 ppm and the same three-point fit.

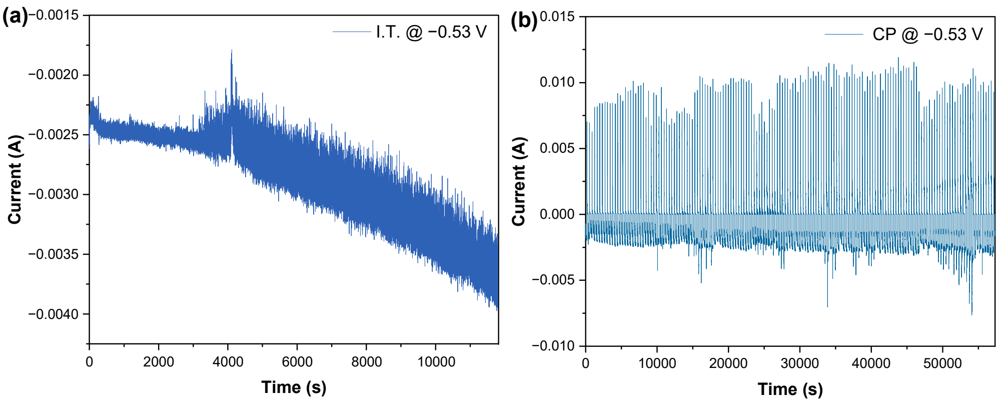

Figure S6. Current–time traces during (a) constant-potential electrolysis at −0.53 V vs. RHE and (b) pulsed electrolysis with E_{red} = −0.53 V and E_{ox} = +0.53 V vs. RHE, each held for 180 s.

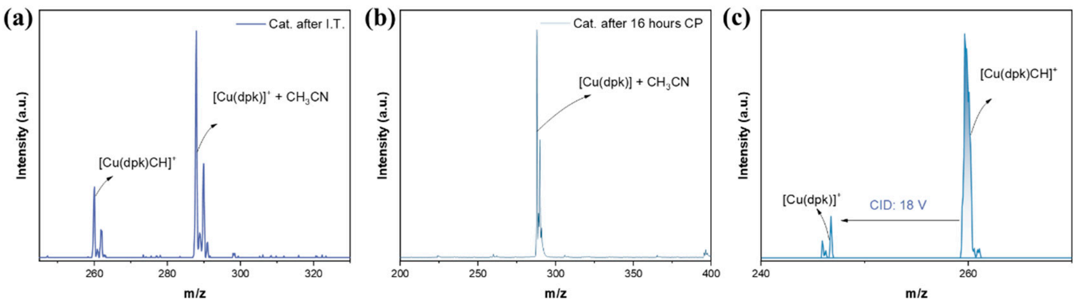

Figure S7. (a) Solution MS after constant-potential electrolysis. (b) Solution MS after approximately 16 h of pulsed electrolysis. (c) CID of the mass-selected m/z = 260 ion, with a fragment at m/z = 247.

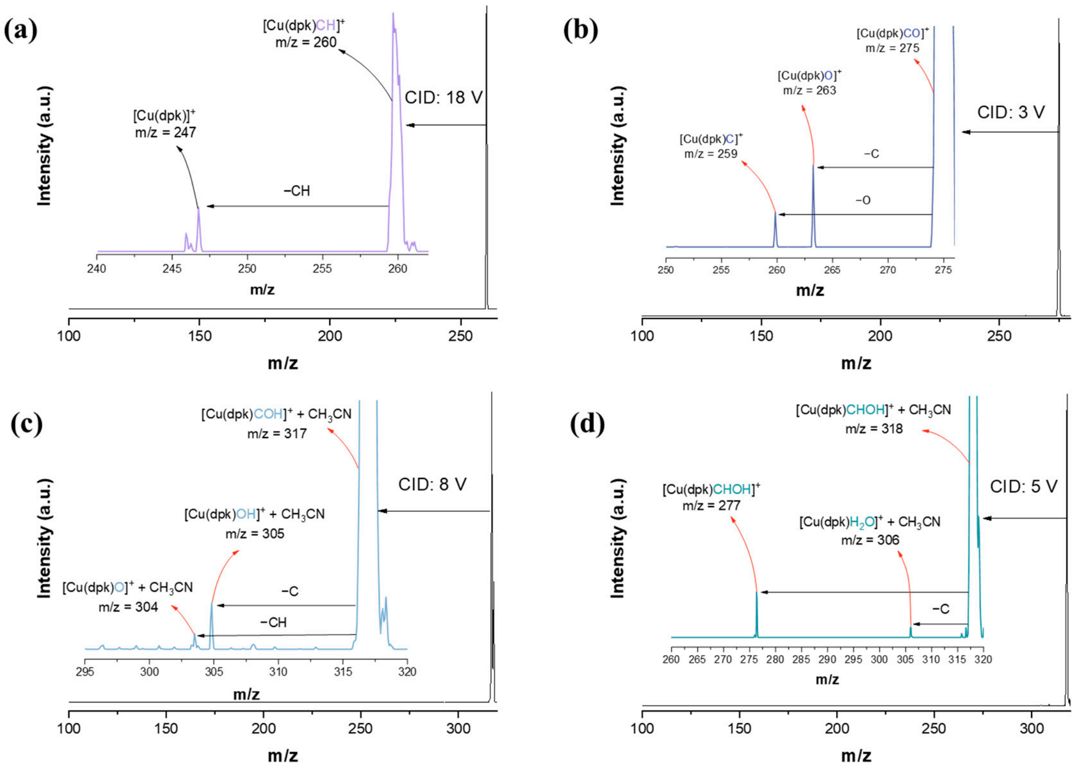

Figure S8. CID spectra supporting the proposed compositions of (a) $[Cu(dpk)CH]^+$, m/z = 260, at 18 V; (b) $[Cu(dpk)CO]^+$, m/z = 275, at 3 V; (c) $[Cu(dpk)COH(CH_3CN)]^+$, m/z = 317, at 8 V; and (d) $[Cu(dpk)CHOH(CH_3CN)]^+$, m/z = 318, at 5 V.

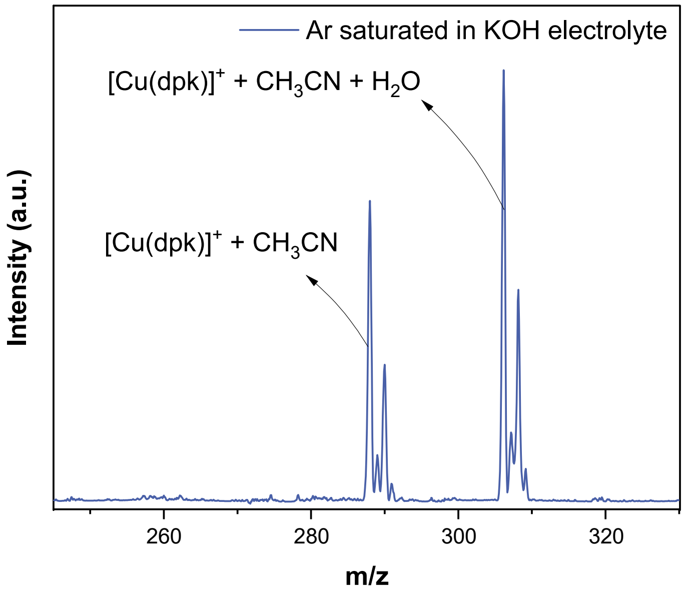

Figure S9. EC-ESI-MS of the catalyst solution in Ar-saturated KOH electrolyte.

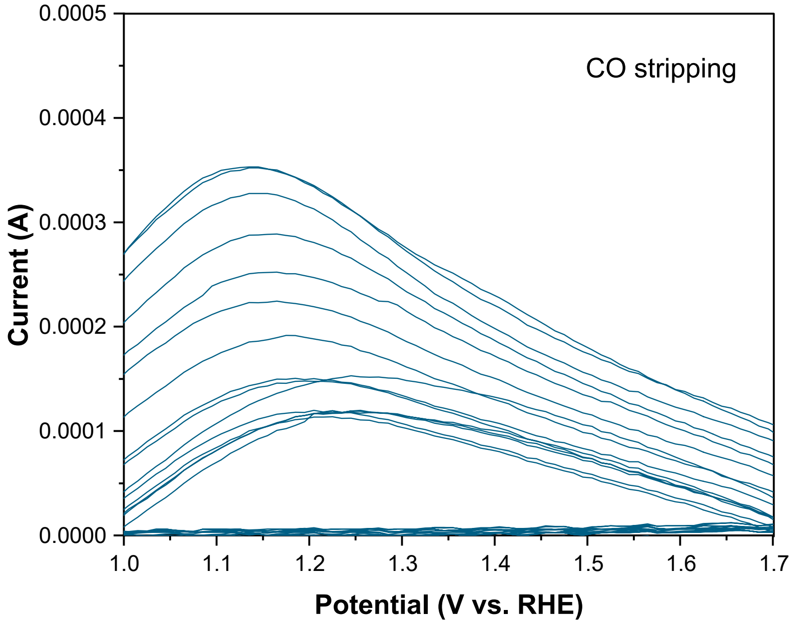

Figure S10. Ar-background-subtracted anodic current during repeated CO-saturated voltammetric scans in dissolved Cudpk catalyst solution. CO and Ar currents were matched at the same potential and scan direction and subtracted pointwise. The displayed segment is 1.0–1.7 V vs. RHE within the full scan window of −1.825 to +3.165 V vs. RHE.

Table S1. The elemental composition determined experimentally and the 6:1 molar mixture composition derived theoretically.

**Element**	**Exp. /%**	**Theo. /%**	**Exp. – Theo**
C	50.21	50.26	−0.05
H	4.17	3.79	+0.38
N	8.72	9.22	−0.50
O	18.69	18.80	−0.11
Cu	18.21	17.93	+0.28

Table S2. Pulse parameters

Parameter	Periodic programme	Finite short sequence
Reduction potential	−0.53 V vs RHE	−0.53 V vs RHE
Oxidation potential	+0.53 V vs RHE	+0.53 V vs RHE
Segment durations	180 s / 180 s	180 s / 180 s
Period and frequency	360 s; 0.00278 Hz	4 pairs + terminal reduction
Reduction duty fraction	50% per complete pair	900/1620 = 55.6%
Segment count	Repeated alternation	5 reduction; 4 oxidation
Total time	16 h = 160 complete pairs	1620 s
Reduction / oxidation time	8 h / 8 h at 16 h	900 s / 720 s
